# Impact of Dietary or Drinking Water *Ruminococcus* sp. Supplementation and/or Heat Stress on Growth, Histopathology, and Bursal Gene Expression of Broilers

**DOI:** 10.3389/fvets.2021.663577

**Published:** 2021-06-29

**Authors:** Adel Hassan Saad, Mohamed S. Ahmed, Mohamed Aboubakr, Hanan A. Ghoneim, Mohamed M. Abdel-Daim, Ghadeer M. Albadrani, Nagah Arafat, Sabreen Ezzat Fadl, Walied Abdo

**Affiliations:** ^1^Nutrition and Clinical Nutrition Department, Faculty of Veterinary Medicine, Matrouh University, Mersa Matruh, Egypt; ^2^Pathology Department, Faculty of Veterinary Medicine, Kafrelsheikh University, Kafr El-Shaikh, Egypt; ^3^Pharmacology Department, Faculty of Veterinary Medicine, Benha University, Banha, Egypt; ^4^Department of Physiology, Faculty of Veterinary Medicine, Damanhour University, Damanhour, Egypt; ^5^Pharmacology Department, Faculty of Veterinary Medicine, Suez Canal University, Ismailia, Egypt; ^6^Department of Biology, College of Science, Princess Nourah Bint Abdulrahman University, Riyadh, Saudi Arabia; ^7^Department of Poultry Diseases, Faculty of Veterinary Medicine, Mansoura University, Mansoura, Egypt; ^8^Biochemistry Department, Faculty of Veterinary Medicine, Matrouh University, Mersa Matruh, Egypt

**Keywords:** heat stress, *Ruminococcus*, enzyme, biochemistry, phagocytic assay, pathology, bursal gene

## Abstract

This research was conducted to evaluate the impact of dietary or drinking water *Ruminococcus* sp. supplementation and/or heat stress (HS) on the growth, serum biochemistry, tissue antioxidant, phagocytic assay, histopathology, and bursa gene expression of broilers. Day-old broiler chicks were allotted into six groups according to HS and/or *Ruminococcus* with or without enzyme supplementation. The first group was the control one, with a formulated diet and normal environmental temperature but without any supplement. The second group fed on *Ruminococcus*-supplemented diet (1 kg/kg diet). The third group fed on a formulated diet without supplement, and *Ruminococcus* and digestive enzymes were given in drinking water (0.1 ml/L). The fourth one was the heat stress group, with a normal formulated diet. The fifth and the sixth groups served as second and third groups, respectively, but with heat stress. The results of this experiment indicated that thermal temperature negatively affected the parameters of growth performance, serum biochemical, tissue antioxidants, and phagocytic assay. Moreover, heat stress led to pathological lesions in the internal organs and affected the expression of some genes related to heat stress, including proapoptotic genes such as caspase8 and bax, inflammatory genes such as NF-κβ1, and heat shock protein such as HSP 70 in the bursal tissue. These bad effects and abnormalities were mitigated by *Ruminococcus* alone or with enzyme supplementation, which improved all the above-mentioned parameters.

## Introduction

Poultry is widely produced and consumed meat worldwide. Its request is expected to continue elevating to meet the animal protein requirement for the ever-growing human population. In the past, it had been common to use antibiotics to promote poultry growth and control intestinal microbiota (1). However, antibiotic growth promoters have side effects on human health due to drug resistance. Over the last 30 years, the way of considering the gastrointestinal tract (GIT) of animals that produce food has been revolutionized (2). This new approach made us conscious of the anatomical system's true complexity in fulfilling digestive, absorptive, metabolic, immunological, and endocrinological roles (3), so gut health is very important in the livestock industry, and researchers (4) are coming up with a new approach in animal science (5). Gut health depends on many factors, including diet, digestion, absorption, immunity, morphology, and microbiota of the GIT (6). In poultry, microorganisms are heavily populated in the GIT and closely and intensively interact with the host and ingested feed. These microorganisms are exogenous and enter the GIT of the bird immediately after hatching; thereafter, it becomes a warm shelter for a complex microbiome consisting primarily of anaerobic bacteria. This intestinal microbiota benefits the host through the supply of nutrients from otherwise poorly used food substrates and regulation of the digestive and immune system function (7). Probiotics and prebiotics ensure the GI microbial community diversity and stability, in addition to favorable interactions with gastroenteric epithelium and the immune system of the host (8). *Ruminococcus* are anaerobic, Gram-positive gut microbes related to the class Clostridia (9). It has a great role in the digestion of fibers in ruminant animals. Thus, it has been found in abundance in health status and markedly depleted in numerous diseases. On the other side, the impact of climate fluctuations in poultry production around the world has become a major challenge. If the global environment changes, the temperature, precipitation levels, and carbon dioxide in the atmosphere change. Farms of poultry primarily depend on weather conditions such as temperature and moisture. Heat stress (HS) is the most important environmental factor affecting poultry farms (10). HS is one of the most important environmental stressors that challenge the global poultry industry. It causes enormous losses in poultry production due to the negative physiological, behavioral, and immunological effects (11). To our knowledge, the detailed pathobiology of heat stress' effect on birds is so far incompletely determined. HS acts on different levels, including genomic, transcriptomic, proteomic, and metabolomics (12). HS was associated with oxidative damages through a decrease in the activity of the mitochondrial respiratory chain with an increase in the reactive oxygen species and, consequently, an increase in lipid peroxidation. Moreover, HS induces inflammation either immediately by the upregulation of cytokine levels or secondary to oxidative stress or disruption of the intestinal integrity and promotion of the invasion of pathogenic bacteria (13). It is noteworthy that HS markedly alters the intestinal microbiota by lowering the content of beneficial bacteria like *Lactobacilli* and *Bifidobacteria* and increasing the pathogenic bacteria such as coliform and *Clostridium* (14, 15).

It was assumed that by using *Ruminococcus* sp. supplementation to the diet or drinking water, it is possible to reduce the effects of heat stress in broiler chicken rearing. For this purpose, the growth performance parameters, hematological parameters, dressing percentage, and total edible carcass were rated. Meanwhile, the enzymatic activity of the liver, selected indicators of antioxidant status and red-ox status in the liver tissue, and phagocytic activity were analyzed in addition to histopathology and some gene expression in the bursal tissue.

## Methods

### Experimental Factors

#### *Ruminococcus* sp.

The GIT microbiota were obtained from Egavet Company, Egypt, including *Ruminococcus* sp. and digestive enzymes (cellulase, xylanase, alpha amylase, and protease). They are digestive enzymes with GIT microbiota obtained from BACTIZAD Company.

#### Experimental Design, Feeding Program, and Management

For the present research, 180 Cobb-505 1-day-old broiler chicks (40–45 g/chick) were obtained from a private farm in the Governorate of Kafrelsheikh. The protocol of the experiment was ethically approved by the Research Ethical Committee of the Faculty of Veterinary Medicine, Kafrelsheikh University, Egypt (approval no. KFS-2019-12). The birds were allotted by ranking method into six groups; each group contained 30 birds in three replicates. The first group (control) was the control one and fed on formulated diet according to NRC (16), without any supplement (Table 1) and with normal environmental temperature for the chicks according to their age (starting with 33°C and then decreasing by 2°C every week until reaching room temperature at 22–25°C) and with light/dark at 23/1 h per day. The second group (R), the intestinal microbiota group, fed on *Ruminococcus*-supplemented diet (1 kg/kg diet). The third group (E), the intestinal microbiota with digestive enzymes group, fed on a formulated diet without supplement, and *Ruminococcus* and digestive enzymes were given in drinking water (0.1 ml/L). The fourth (HS) one was the heat stress group and fed on a formulated diet without any supplement but exposed to heat stress (38, 35, and 32–33°C for the 1st, 2nd, and 3rd to 6th weeks, respectively). The fifth (R+HS) and the sixth (E+HS) groups served as second and third groups, respectively, but with heat stress. All groups of chicks were kept in a clean room with good ventilation and vaccinated at 7–12 days against Newcastle disease and infectious bursal disease, respectively. Food and water were added *ad libitum* to the chicks, and they were maintained for 42 days under good sanitation and hygiene.

**Table 1:** Ingredients and calculated chemical composition of basal diet.

**Physical composition**	**Basal diet (0–3) weeks**	**Basal diet (3–6) weeks**
Yellow corn	55.38	60
Soybean meal, 44%	27	28
Corn gluten, 62%	9.5	4.2
Corn oil	4	4.19
Dicalcium phosphate	1.75	1.25
Lime stone	1.27	1.4
Lysine	0.07	0.05
Methionine	0.11	0.04
Choline, 60%	0.22	0.17
Common salt	0.4	0.4
Premix^a^	0.3	0.3
**Chemical composition, %**		
ME kcal/kg	3215.4	3207.9
Crude protein	23	20.1
Calcium	1	0.9
Available phosphorus	0.45	0.35
Lysine	1.1	1
Methionine + cysteine	0.9	0.72
Choline	1,300 mg/kg	1,000 mg/kg

a *The premix (Multivita Co.) used composed of vitamin A = 12,000,000 IU, vitamin D3 = 2,200,000 IU, vitamin E = 10,000 mg, vitamin K3 = 2,000 mg, vitamin B1 = 1,000 mg, vitamin B2 = 5,000 mg, vitamin B6 = 1,500 mg, vitamin B12 = 10 mg, niacin = 30,000 mg, biotin = 50 mg, folic acid = 1,000 mg, pantothenic acid = 10,000 mg, iron = 30,000 mg, manganese = 60,000 mg, copper = 4,000 mg, zinc = 50,000 mg, iodine = 1,000 mg, cobalt = 100 mg, selenium = 100 mg, and calcium carbonate (CaCO_3_) carrier to 3,000 g*.

The feed proximate analysis was done (17), and the weight of the birds was measured individually at the start of the experiment and every week. Then, all the production parameters were calculated, including weight of the body (BW) (18), weight gain (19), and feed conversion ratio (FCR) (20).

#### Dressing Percentage and Total Edible Carcass (%)

At the termination of the growing period (42 days), three broilers were taken randomly from each replicate (nine birds/group), deprived of food for 12 h, and then weighed individually. After that, the birds were euthanized by using an overdose of anesthesia and slaughtered to complete the bleeding, followed by plucking of the feathers. The dressed weight was taken after the removal of the head, viscera, shanks, gizzard, liver, heart, and immune organs. The dressing percentage was calculated on the basis of live weight, where dressing percentage was defined as carcass weight divided by the final live weight record of the bird.

#### Blood and Tissue Samples

The birds for the sample collection were randomly selected and anesthetized by an intraperitoneal injection of sodium pentobarbital (50 mg/kg). Two samples from each bird (5 ml blood/sample/bird) were collected from the wing vein (six samples/group). The first sample was collected with an anticoagulant for hematological examination and phagocytic assay and the second one without anticoagulant for serum separation. The serum activities of aspartate transaminase (AST), alanine transaminase (ALT), and alkaline phosphatase (ALP) were measured by using commercial kits obtained from BIODIAGNOSTIC Company, Giza, Egypt.

After the sample collection, the birds were euthanized by using an overdose of the anesthesia for tissue sample collection. The tissue samples were bursal sample (six samples/group) for gene expression and kept at −80°C until use, liver sample (six samples/group) for the activities of antioxidant enzymes [catalase (CAT), superoxide dismutase (SOD), and glutathione peroxidase (GPx)], and malondialdehyde (MDA) concentration and liver, spleen, and intestine for histopathology.

#### Selected Indicators of Antioxidant Status and Red-Ox Status in Liver Tissue

Each liver sample (1 g) was homogenized with 5 ml phosphate buffer, pH 7.4, on ice using an electrical homogenizer. Tissue homogenate was centrifuged at 1,200 × *g* for 20 min at 4°C for the separation of supernatants. The supernatant was used for determining the activities of antioxidant enzymes (CAT, SOD, and GPx) and MDA concentration.

Briefly, CAT activity was assessed according to the method described by Aebi (21) and expressed as microns per gram of tissue. Determination of SOD was done following the instructions of the kits (Biodiagnostic, # SD 2521, Egypt). The increase of the absorbance was monitored at 560 nm over 5 min and expressed as microns per gram (22). GPx was determined according to the instructions of the Biodiagnostic kit (Biodiagnostic, #GP 2524, Egypt). The decrease of absorbance was recorded at 340 nm over a period of 3 min and expressed as microns per gram (23). For determination of MDA, the manufacturer's protocol of Biodiagnostic kit was followed (Biodiagnostic, #MD 2529, Egypt), measured spectrophotometrically at 534 nm, and expressed as nanomole per gram of tissue (24).

#### Phagocytic Assay

Analyses of phagocytic activity and index were done according to the approach mentioned by Kawahara et al. (25) and El-Kassas et al. (26) using *Candida albicans*. Equal volumes of fresh heparinized blood sample, *C. albicans* suspension (equivalent to 1 × 106), and fetal bovine serum were mixed and incubated at 37°C for 30 min. The samples were centrifuged at 1,500 rpm for 10 min, and then the sediment was resuspended on a glass slide. The slides were then fixed with methanol for further staining with polychrome methylene blue and eosin stain. The phagocytic activity (PA) and phagocytic index (PI) were determined. PA was calculated as the percentage of phagocytic cells that engulfed yeast cells, while PI was assessed as the total number of yeast cells phagocytized per phagocytic cell.

#### Histopathological Investigation

Hepatic, intestinal, and spleen sections have been taken after necropsy and immediately fixed in 10% buffered formalin and treated with the routine paraffin embedding portion for histopathological evaluation. Then, 3-μm-thick sections were cut and stained using H&E (27).

#### Bursal Gene Expression

For complete RNA extraction, bursal samples were used with easy RED total RNA extraction kits (iNtRON Biotechnology, Inc.) as per the instructions defined by the manufacturer. The completeness of the RNA was tested using the electrophoresis of agarose gel. The first cDNA branch was synthesized to complete the RNA using the cDNA synthesis kit Intron-Power (cat. no. 25011).

To amplify the selected chicken (*Gallus gallus*) genes, specific primers were used with GAPDH as housekeeping (internal standard) gene primer sequence (Table 2). The qRT-PCR assay was carried out using a Stratagene MX300P Q-PCR system (Agilent Technologies), using Real MODTM Green FAST qPCR master mix (S) following the manufacturer's recommendations. MxPro QPCR Software was used for data collection.

**Table 2:** Primer sequences (5′-3′) used in real-time PCR.

**Gene**	**Primer**	**References**
β-actin	F: ACCTGAGCGCAAGTACTCTGTCT R: CATCGTACTCCTGCTTGCTGAT	NM_205518.1 (28 )
CASP8	F: CTGAAACTACAATGCCGGACG R: GGCTCTTGTCCACTTTCCCA	NM_204592 (29)
BAX	F: TCCTCATCGCCATGCTCAT R: CCTTGGTCTGGAAGCAGAAGA	XM_422067 (30)
NF-κβ1	F: TACCGGGAACAACACCACTG R: CAGAGGGCCTTGTGACAGTA	NM_205134 (29)
HSP70	F: CCAAGAACCAAGTGGCAATGAA R: CATACTTGCGGCCGATGAGA	EU747335 (31)

The relative gene expression levels were evaluated using the 2^−Δ*Δct*^ method (32).

### Statistical Analysis

The achieved results were statistically analyzed by one-way ANOVA using GRAPPAD prism, version 5. A comparison between the groups was performed by Tukey's *post-hoc* test. Data were expressed as mean ± SD.

## Results

### Clinical Signs and Postmortem Lesions of Heat Stress

Experimental heat stress in broilers appeared as some clinical signs and postmortem (PM) lesions in the form of panting, increased water intake (thirst), diarrhea, reduced feed consumption, legs and wings outstretched, and prostration. On the other side, the PM lesions were manifested as congested carcass and mucoid exudate in the nostrils and mouth. The above-mentioned changes were decreased in the treated groups (Table 3).

**Table 3:** Effects of supplementation with digestive enzymes and/or *Ruminococcus* sp. on the growth performance of broiler chicken under heat stress (at day 42), *n* = 30.

**Parameters**	**Groups**					
	**Control**	***R***	***E***	**HS**	**HS+R**	**HS+E**
Initial body weight (g/chick)	49.58 ± 0.15^a^	47.92 ± 0.48^a^	48.33 ± 0.33^a^	48.67 ± 0.38^a^	48.75 ± 0.30^a^	48.33 ± 0.31^a^
Final body weight (g/bird)	2,494 ± 2.08^c^	2,516.67 ± 1.45^b^	2,589.33 ± 0.67^a^	2,084.67 ± 2.91^f^	2,441.33 ± 2.40^d^	2,354 ± 2.08^e^
Total weight gain (g/bird)	2,444.33 ± 1.86^c^	2,468.00 ± 2.31^b^	2,540.33 ± 0.88^a^	2,035.33 ± 2.40^f^	2,339.00 ± 2.08^d^	2,306.00 ± 2.65^e^
FCR	1.56 ± 0.00^b^	1.46 ± 0.02^d^	1.34 ± 0.01^e^	1.79 ± 0.01^a^	1.51 ± 0.00^c^	1.48 ± 0.01^cd^
Feed intake (g/bird)	3,810.15 ± 1.05^a^	3,608.28 ± 1.08^b^	3,409.04 ± 0.09^d^	3,635.24 ± 2.00^b^	3536.89 ± 1.00^c^	3,417.88 ± 1.07^d^
Number of dead	1	0	0	2	0	2
Survival, %	96.67	100	100	93.33	100	93.33
Mortality, %	3.33	0	0	6.67	0	6.67

*Values are expressed as mean ± standard errors. Means in the same row with different superscript letters (a–f) significantly differ at p ≤ 0.05*.

### Growth Performance

Dietary supplementation of digestive enzymes and/or *Ruminococcus* sp. significantly (*P* ≤ 0.05) increased the BW and total weight gain and decreased the FCR compared to the control group (Table 3). On the other side, the above-mentioned parameters were badly affected in the HS group as compared to the other groups. Moreover, body weight, weight gain, and FCR were improved significantly in heat stress groups that were supplemented with digestive enzymes and/or *Ruminococcus* sp. On the other side, the feed intake was significantly (*P* ≤ 0.05) decreased in the HS group compared to the control group, which was improved with treatments.

### Dressing Percentage and Total Edible Carcass (%)

Table 4 shows the effect of digestive enzymes and/or *Ruminococcus* sp. on some carcass traits of broilers. There were significant (*P* ≤ 0.05) and insignificant (*P* ≤ 0.05) increases in dressing and gizzard and heart percent, respectively, in the group that was supplemented with digestive enzymes and/or *Ruminococcus* sp. without heat stress compared to the control and R groups. Moreover, the thermal temperature significantly (*P* ≤ 0.05) decreased liver percent compared to the other groups. Meanwhile, the immune organs, including the thymus and bursa, and spleen were insignificantly and significantly (*P* ≤ 0.05) decreased, respectively, in the HS group compared to the other groups.

**Table 4:** Effects of supplementation with digestive enzymes and/or *Ruminococcus* sp. on the results of the slaughter analysis of chickens (at day 42) and relative weights of selected organs as percentage of body weight.

**Parameters, %**	**Groups**					
	**Control**	***R***	***E***	**HS**	**HS+R**	**HS+E**
Dressing	75.83 ± 1.11^b^	76.64 ± 1.12^b^	87.59 ± 0.55^a^	72.59 ± 1.56^c^	75.78 ± 1.58^b^	76.60 ± 0.86^b^
Gizzard	0.93 ± 0.16^b^	1.06 ± 0.07^b^	1.44 ± 0.01^a^	0.29 ± 0.03^c^	1.00 ± 0.06^b^	1.07 ± 0.12^b^
Heart	0.49 ± 0.06^ab^	0.47 ± 0.06^ab^	0.58 ± 0.07^a^	0.39 ± 0.03^b^	0.45 ± 0.02^ab^	0.46 ± 0.03^ab^
Liver	1.98 ± 0.11^ab^	1.86 ± 0.09^b^	2.43 ± 0.29^a^	1.43 ± 0.29^c^	2.22 ± 0.23^ab^	2.07 ± 0.12^ab^
Abdominal fat	0.81.15 ± 0.05^b^	1.05 ± 0.16^b^	0.80 ± 0.02^b^	1.09 ± 0.06^b^	1.76 ± 0.32^a^	1.04 ± 0.18^b^
Thymus	0.38 ± 0.02	0.38 ± 0.03	0.34 ± 0.14	0.29 ± 0.03	0.26 ± 0.01	0.33 ± 0.02
Spleen	0.19 ± 0.01^a^	0.19 ± 0.00^a^	0.18 ± 0.02^a^	0.09 ± 0.00^b^	0.17 ± 0.02^a^	0.16 ± 0.01^a^
Bursa	0.09 ± 0.01	0.09 ± 0.03	0.09 ± 0.03	0.06 ± 0.02	0.07 ± 0.01	0.12 ± 0.03
Proventriculus	0.58 ± 0.14	0.37 ± 0.04	0.42 ± 0.05	0.43 ± 0.04	0.47 ± 0.02	0.68 ± 0.11

*Values are expressed as mean ± standard errors. Means in the same row with different superscript letters (a–c) significantly differ at p ≤ 0.05*.

### Hematological Parameters

Table 5 shows the hematological parameters of broilers supplemented with digestive enzymes and/or *Ruminococcus* sp. There were insignificant (*P* ≤ 0.05) increases in red blood cell count and hemoglobin percent in groups that were supplemented with digestive enzymes and/or *Ruminococcus* sp. without heat stress compared to the control group. Moreover, the previous measurements were significantly (*P* ≤ 0.05) decreased in the HS group compared to the groups that were supplemented with digestive enzymes and/or *Ruminococcus* sp. with heat stress. On the other hand, the hematocrit percent was increased in the *Ruminococcus* sp. group (R) insignificantly and significantly (*P* ≤ 0.05) compared to the control and the group that received digestive enzymes and *Ruminococcus* sp. (E group). without heat stress, respectively. Meanwhile, the hematocrit percent was significantly (*P* ≤ 0.05) decreased in heat stress groups compared to the other groups without heat stress.

**Table 5:** Effects of supplementation with digestive enzymes and/or *Ruminococcus* sp. on the hematological parameters of broiler chicken under heat stress (at day 42).

**Parameters**	**Groups**					
	**Control**	***R***	***E***	**HS**	**HS+R**	**HS+E**
RBC count (×10^6^/μl)	3.39 ± 0.12^a^	3.59 ± 0.17^a^	3.74 ± 0.23^a^	2.07 ± 0.13^c^	2.36 ± 0.14^bc^	2.60 ± 0.17^b^
Hb (g/dl)	10.17 ± 0.35^a^	10.77 ± 0.52^a^	11.23 ± 0.69^a^	6.20 ± 0.40^c^	7.07 ± 0.41^bc^	7.80 ± 0.50^b^
HCT (%)	40.67 ± 1.41^ab^	43.07 ± 2.08^a^	34.27 ± 3.47^bc^	24.80 ± 1.62^d^	28.27 ± 1.62^cd^	31.20 ± 2.01^cd^
WBC count (×10^3^/μl)	7,333.67 ± 2.03^f^	7,937.67 ± 1.86^e^	8,223.33 ± 2.03^d^	15,503.33 ± 2.60^a^	10,385.67 ± 2.85^b^	9,778 ± 0.58^c^
LYM (×10^3^/μl)	72.33 ± 0.33^a^	72.00 ± 2.52^a^	74.33 ± 2.73^a^	56.00 ± 3.06^b^	70.33 ± 2.96^a^	68.33 ± 2.03^a^
HET (×10^3^/μl)	12.67 ± 1.67^b^	12.67 ± 0.67^b^	13.33 ± 1.33^b^	27.67 ± 2.96^a^	16.33 ± 2.60^b^	16.00 ± 1.53^b^
ESI (×10^3^/μl)	13.33 ± 1.20^a^	13.33 ± 3.18^a^	11.00 ± 1.15^a^	12.33 ± 0.33^a^	11.33 ± 0.88	12.67 ± 0.67^a^
MON (×10^3^/μl)	1.33± 0.33^a^	1.67 ± 0.33^a^	1.33 ±0.33^a^	1.67 ± 0.33^a^	1.33± 0.33^a^	2.00 ± 0.58^a^
BAS (×10^3^/μl)	0.33± 0.33^bc^	0.33 ± 0.33^bc^	0.00 ± 0.00^c^	2.33 ± 0.33^a^	0.67 ± 0.33^bc^	1.00 ± 0.00^b^

*Values are expressed as mean ± standard errors. Means in the same row with different superscript letters (a–f) significantly differ at p ≤ 0.05*.

*RBCs, red blood cells; Hb, hemoglobin; HCT, hematocrit; WBCs, white blood cells; LYM, lymphocyte; HET, heterophil; ESI, eosinophil; MON, monocytes; BAS, basophil*.

Regarding the results of the leukogram, there was a significant (*P* ≤ 0.05) increase in the total leukocytic count in the HS group compared to the other groups with or without heat stress. This decrease was improved with treatments even with heat stress. Moreover, the lymphocyte and heterophils were significantly (*P* ≤ 0.05) decreased and increased, respectively, in the HS group when compared to the other groups with or without heat stress. Meanwhile, the eosinophil and monocyte levels were not affected by heat stress or treatments. On the other hand, the basophil level was significantly (*P* ≤ 0.05) increased in the HS group when compared to the other groups with or without heat stress.

### Enzymatic Activity of the Liver

Table 6 shows the serum enzymes related to liver function. There was a significant (*P* ≤ 0.05) increase in the activities of the liver enzymes (ALT, AST, and ALP) enzymes in the HS group compared to the other groups. Moreover, the previous enzymes were significantly (*P* ≤ 0.05) increased in heat stress groups and supplemented with digestive enzymes and/or *Ruminococcus* sp. compared with the control group and groups that were supplemented with digestive enzymes and/or *Ruminococcus* sp. without heat stress.

**Table 6:** Effects of supplementation with digestive enzymes and/or *Ruminococcus* sp. on the liver enzyme activity of chickens' blood serum at 42 days.

**Parameters**	**Groups**					
	**Control**	***R***	***E***	**HS**	**HS+R**	**HS+E**
ALT (u/L)	10.67 ± 0.88^cd^	8.33 ± 0.88^d^	10.67 ± 1.45^cd^	17.33 ± 1.76^a^	15.33 ± 0.33^ab^	13.33 ± 0.88^bc^
AST (u/L)	176.00 ± 2.08^d^	189.33 ± 1.20^c^	170.67 ± 1.20^d^	268.67 ± 2.73^a^	230.67 ± 1.20^b^	190.33 ± 2.40^c^
ALP (u/L)	538.67 ± 2.03^e^	616.33 ± 2.33^d^	421.33 ± 1.20^f^	1,481.67 ± 2.19^a^	1,092.00 ± 3.51^b^	797.67 ± 1.45^c^

*Values are expressed as mean ± standard errors. Means in the same row with different superscript letters (a–f) significantly differ at p ≤ 0.05*.

*ALT, alanine transaminase; AST, aspartate transaminase; ALP, alkaline phosphatase*.

### Selected Indicators of Antioxidant Status and Red-Ox Status in Liver Tissue

The level of MDA in liver tissue was significantly (*P* ≤ 0.05) increased in the HS group compared to the other groups (Table 7). This elevation in the MDA level was mitigated by digestive enzymes and/or *Ruminococcus* sp. in the heat stress groups. On the other hand, tissue SOD and GPx activities were significantly and insignificantly (*P* ≤ 0.05) increased, respectively, in the group that was supplemented with digestive enzymes and *Ruminococcus* sp. (E group) without heat stress compared to the control group and group that received *Ruminococcus* sp. (R group) only without heat stress or digestive enzymes. Meanwhile, tissue CAT activity was insignificantly (*P* ≤ 0.05) increased in the group that was supplemented with *Ruminococcus* sp. (R) without digestive enzymes and heat stress compared to the control group and group that received digestive enzymes and *Ruminococcus* sp. (E) without heat stress. Moreover, all antioxidant enzyme activities were improved with a digestive enzyme and *Ruminococcus* sp. supplementation (E).

**Table 7:** Effects of supplementation with digestive enzymes and/or *Ruminococcus* sp. on the liver tissue antioxidants and peroxide of chickens' blood serum at 42 days.

**Parameters**	**Groups**					
	**Control**	***R***	***E***	**HS**	**HS+R**	**HS+E**
MDA (n mol/g)	12.65 ± 0.02^c^	11.98 ± 0.08^cd^	10.65 ± 0.01^d^	25.8 ± 0.03^a^	19.8 ± 0.01^b^	18.00 ± 0.04^b^
CAT (u/g)	44.59 ± 2.29^ab^	48.34 ± 4.37^a^	46.49 ± 2.67^ab^	28.61 ± 1.54^d^	34.12 ± 3.17^cd^	38.79 ± 2.48^bc^
SOD (u/g)	931.67 ± 1.45^d^	940.33 ± 2.60^c^	1,142.33 ± 2.91^a^	448.18 ±1.45^f^	830.52 ± 1.48^e^	970.67 ± 2.33^b^
GPx (u/g)	54.75 ± 3.21^ab^	53.29 ± 1.06^ab^	61.23 ± 2.83^a^	21.66 ±4.34^d^	42.89 ± 2.99^c^	49.97 ± 2.35^bc^

*Values are expressed as mean ± standard errors. Means in the same row with different superscript letters (a–f) differ at p ≤ 0.05*.

*MDA, malondialdehyde; CAT, catalase; SOD, superoxide dismutase; GPx, glutathione peroxidase*.

### Phagocytic Assay

**Figure 1:**
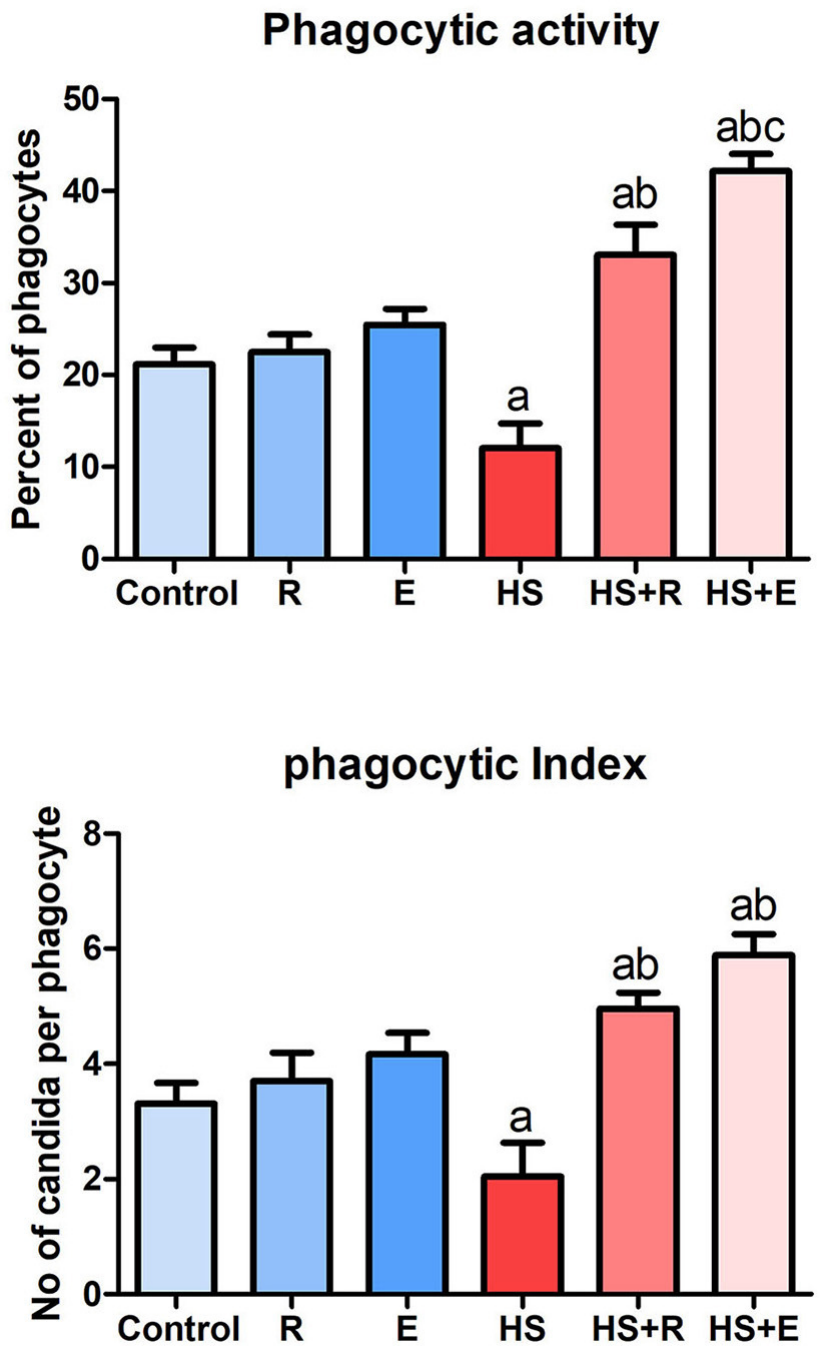
Phagocytic activity and index of broiler chicks.

Both phagocytic activity and index (Figure 1) were markedly decreased in HS birds in comparison with the control birds (*P* > 0.005), while supplementation of heat-stressed birds with *Ruminococcus* sp., in both powder and liquid forms, showed a marked increase of phagocytic activity and index in comparison with the HS group (*P* > 0.005). Supplementation of the liquid form (*E*) was associated with a significant increase of phagocytic activity than of the powder form (*R*) of *Ruminococcus* (*P* > 0.05).

### Pathological Findings of Broiler Chickens

**Figure 2:**
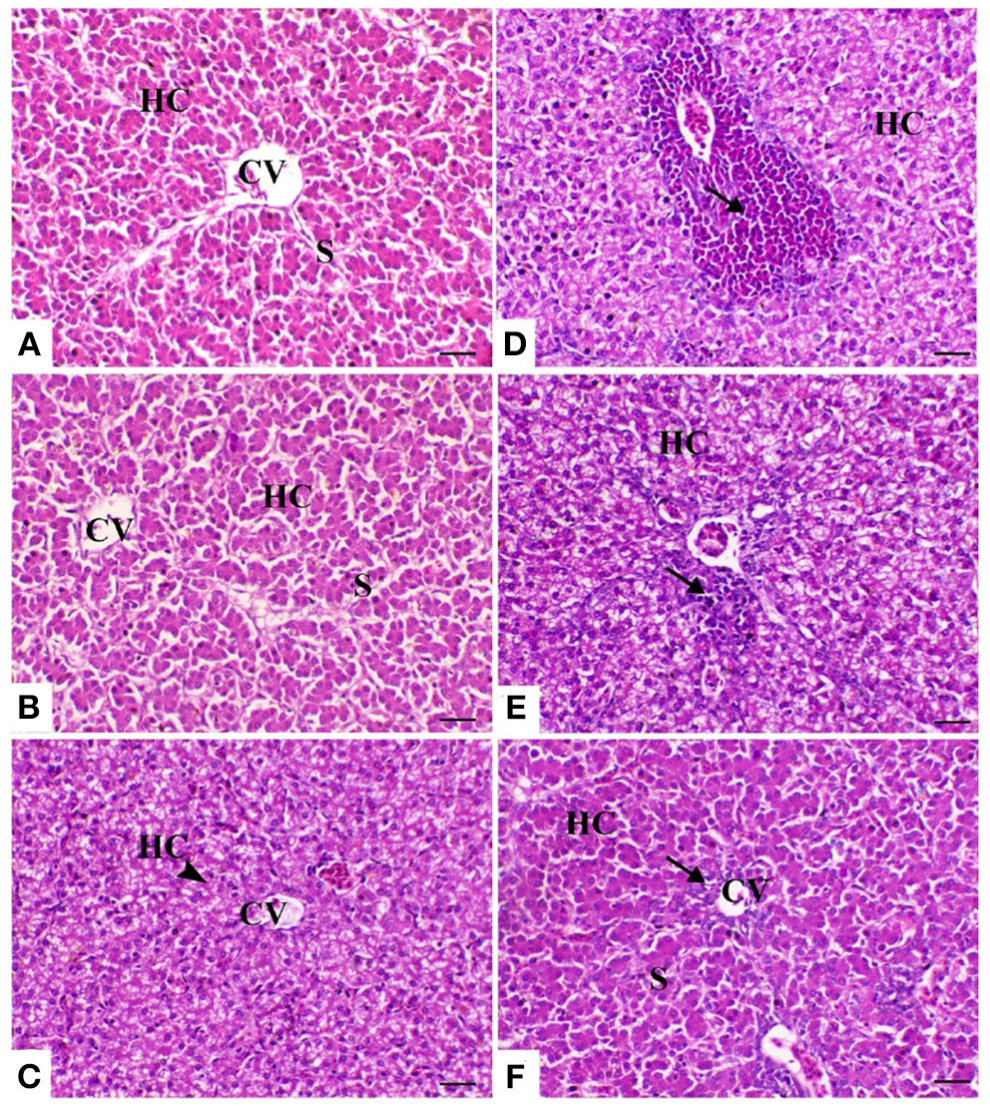
Hepatic sections of the different treated groups. **(A)** Control group. CV, central vein; HC, hepatocytes; S, blood sinusoids. **(B)** Sham group R (the arrowhead indicates normal hepatocytes). **(C)** Sham group E. **(D)** Heat stress (HS) group (the arrow indicates periportal heterophilic infiltration). **(E)** HS+R (the arrow indicates mild periportal mononuclear cell infiltration). **(F)** HS+E (the arrow indicates a slight regeneration of the bile duct lining epithelium and mild inflammatory cell infiltration). H&E, ×200, bar = 50 μm.

The liver of the control birds showed a normal pattern of the hepatic lobule with a centrally located central vein and the arrangement of hepatocytes in cords that were separated from each other by hepatic sinusoids. The liver of birds treated with powder *Ruminococcus* (*R*) showed the same morphology as that in the control group. Meanwhile, there was obvious cytoplasmic vacuolation observed in birds supplemented with the liquid form of *Ruminococcus* (*E*). This vacuolation was consistent with mild glycogen storage. The liver of birds exposed to heat stress was severely affected and showed massive degeneration of hepatocytes as fatty degeneration and heterophilic infiltration. In heat-stressed birds treated with powder (*R*) *Ruminococcus*, the liver showed a marked decrease of hepatic vacuolation of hepatocytes with mild mononuclear cell infiltration. In heat-stressed birds treated with liquid (*E*) *Ruminococcus*, the liver showed normal hepatocytes with slight periportal mononuclear cell infiltration (Figure 2).

**Figure 3:**
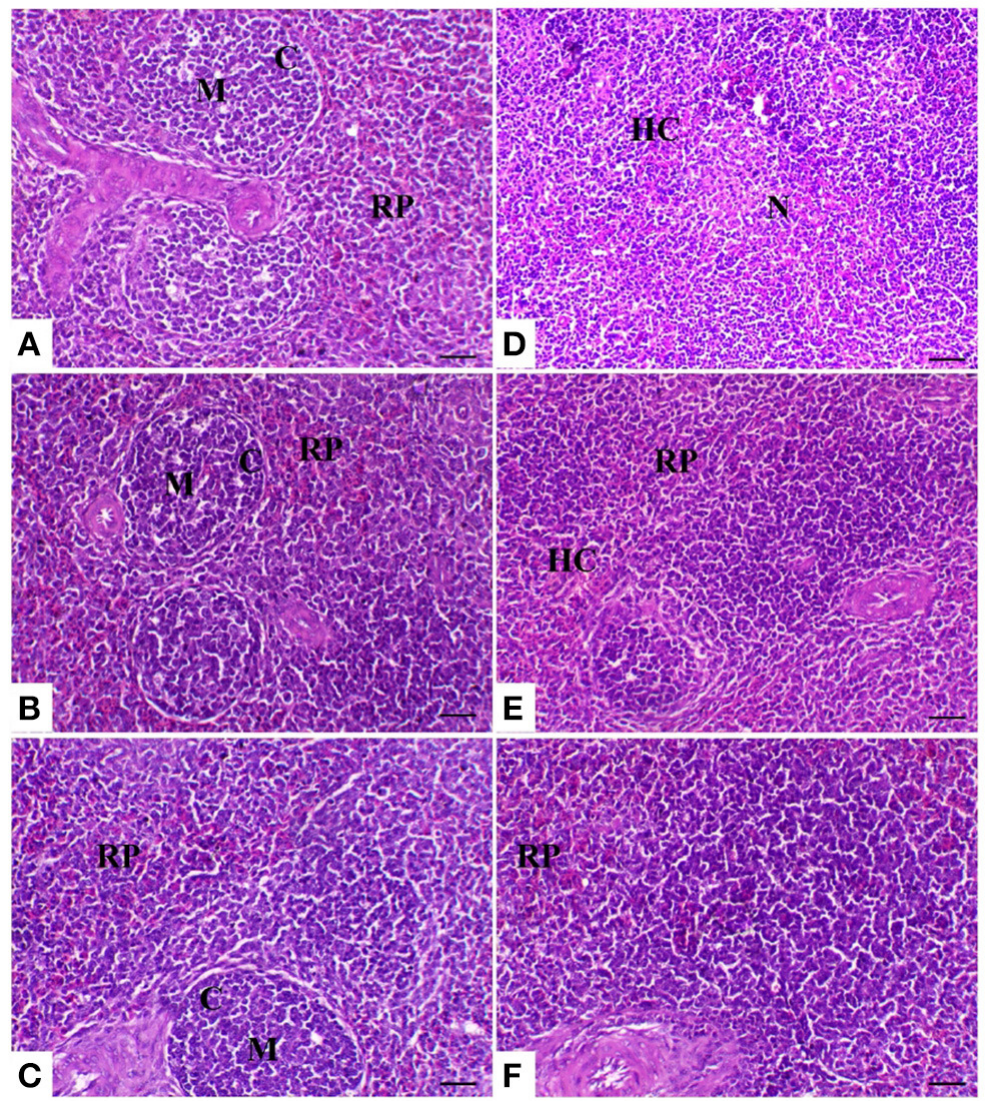
Splenic sections of the different treated groups. **(A)** Spleen of the control group showing normal lymphoid follicles (M and C indicate the medulla and cortex of the lymphoid follicle, respectively). Sham groups **(B)** R and **(C)** E revealing normal lymphoid follicles. **(D)** Spleen of the heat stress (HS) group showing marked lymphoid necrosis and histocytic cell proliferation. **(E)** Spleen of the HS+R showing a marked decrease of histocytes and a slight degree of lymphoid depletion. **(F)** Spleen of the HS+E showing a marked increase of lymphoid elements within the follicle. H&E, ×200, bar = 50 μm.

The spleen of control birds showed distinct white and red pulps. The white pulp was composed of small-, medium-, and large-sized lymphocytes and plasma cells with intact arteriole. The red pulp was composed of venous sinuses with various types of cells. The spleen of birds treated with powder (*R*) and liquid (*E*) forms of *Ruminococcus* showed a nearly similar morphology to that noticed in the control group. The spleen of birds exposed to heat stress was severely affected and showed marked lymphoid depletion and necrosis with massive histiocytic infiltration and complete absence of the normal architectural pattern of the spleen. In heat-stressed birds treated with powder (*R*) *Ruminococcus*, the spleen showed normal morphology with white and red pulp and slight lymphocytic depletion and histiocytic infiltration; however, in heat-stressed birds treated with liquid (*E*) *Ruminococcus*, the spleen showed mild lymphocytic depletion (Figure 3).

**Figure 4:**
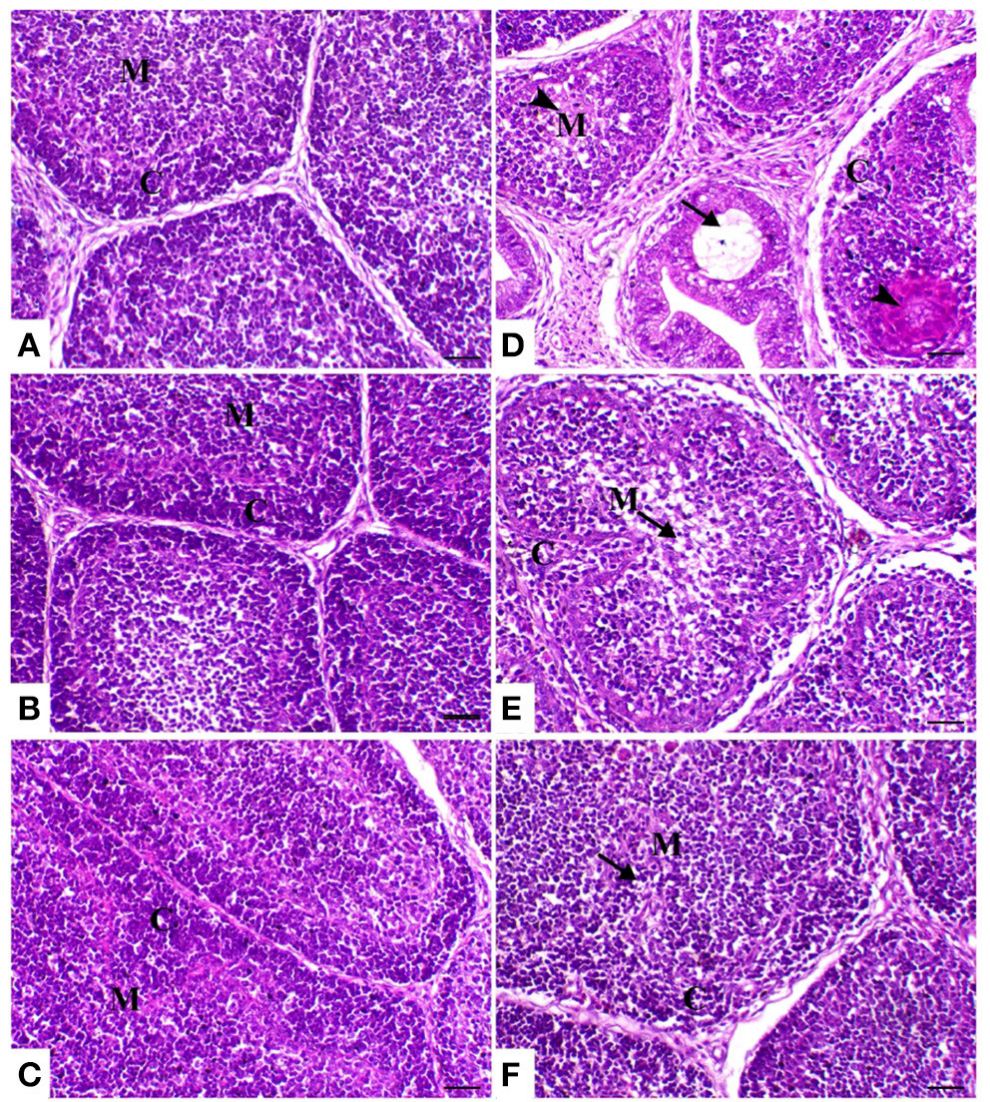
Bursa of Fabricius sections of the different treated groups. **(A)** Spleen of the control group showing normal lymphoid follicles separated with slight fibrous connective tissue septa (M and C indicate the medulla and cortex of the lymphoid follicle, respectively). Sham groups **(B)** R and **(C)** E revealing normal lymphoid follicles. **(D)** Spleen of heat stress (HS) group showing marked atrophy of the lymphoid follicle with medullary necrosis (arrowheads), interstitial fibrosis, and mucosal cyst formation (arrow). **(E)** Spleen of HS+R showing medullary lymphoid depletion (arrow). **(F)** Spleen of HS+E showing a marked restoration of lymphoid content within the follicle. H&E, ×200, bar = 50 μm.

In the control group birds, the bursal follicles had a darkly stained peripheral cortex composed mainly of closely packed lymphocytes and a pale-stained central medulla containing fewer cells of different sizes. The bursal follicles were separated from the adjacent follicles by connective tissue fibers containing blood vessels and a few cells. In the both powder (*R*) and liquid (*E*) *Ruminococcus*-treated group, the bursal follicle remained in the same normal structure as the control ones, but with less cellularity in the medulla. In the heat stress-treated group, the bursal follicles showed a small-sized cortex with slightly packed lymphocytes, medullary necrosis, and hyalinization. Microcysts were observed in the mucosal folds. There was interstitial edema with increased thickness of the interfollicular connective tissue. In heat-stressed birds treated with powder *Ruminococcus* (HS+R), the bursal follicles were well-formed, with intact cortex and less cellularity in the medulla. Meanwhile, in heat-stressed birds treated with liquid *Ruminococcus* (HS+E), the bursal follicles were completely filled with lymphoid cells (Figure 4).

**Figure 5:**
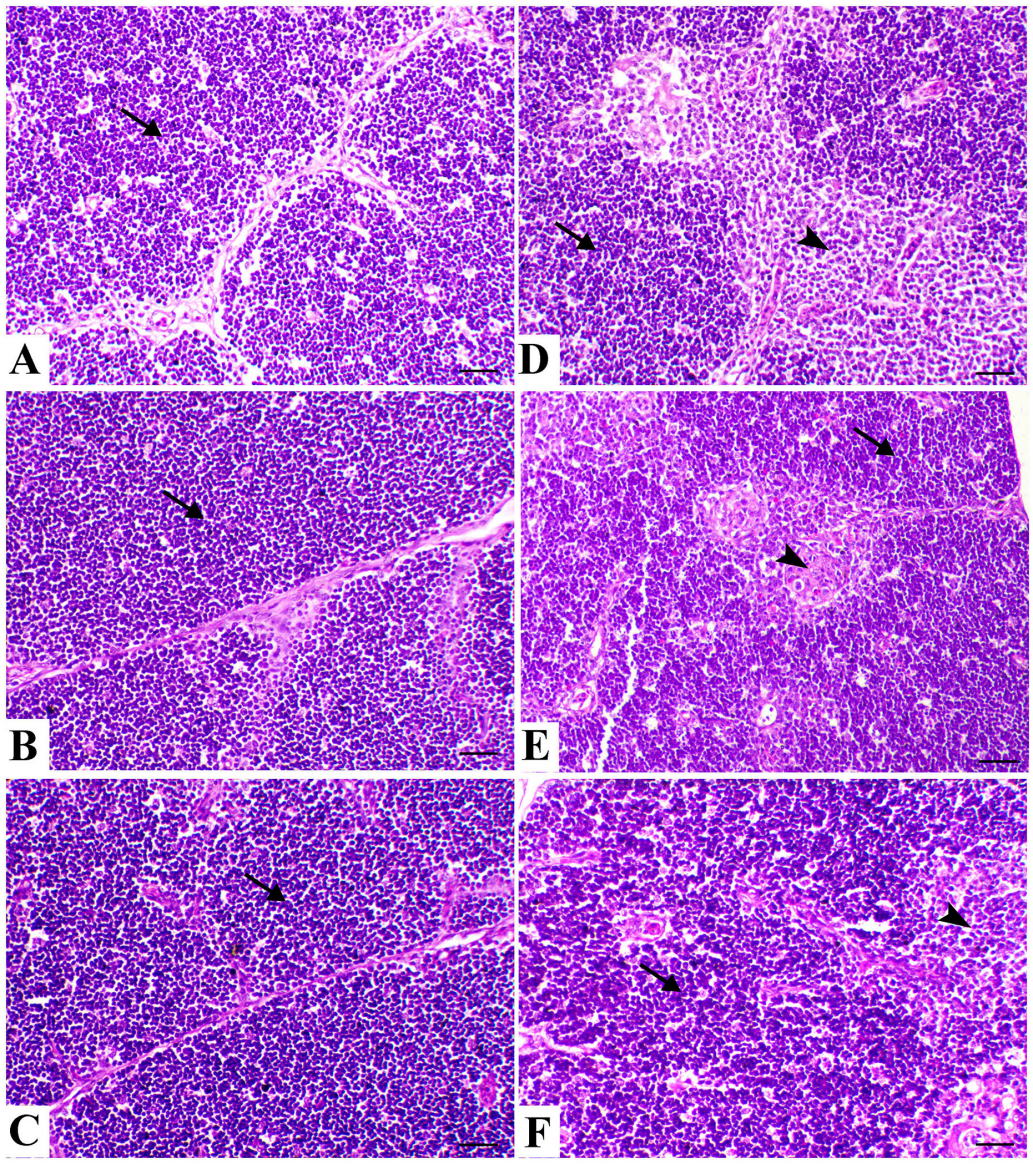
Thymus sections of the different treated groups. **(A)** Thymus of the control group showing normal thymic compartments (the arrow indicates normal thymocytes). Sham groups **(B)** R and **(C)** E revealing normal thymic compartments. **(D)** Spleen of the heat stress (HS) group showing marked thymic depletion (arrow). **(E)** Spleen of HS+R showing medullary lymphoid depletion (arrowhead). **(F)** Spleen of HS+E showing a marked restoration of lymphoid content within the follicle. H&E, ×200, bar = 50 μm.

The thymic compartments of the control birds showed an outer deeply stained cortex containing a large number of small lymphocytes with a large nucleus and a central lightly stained medulla containing many epithelial cells with a large pale nucleus and few lymphocytes. The thymic compartments were separated from each other by interstitial connective tissue septa. In both powder (*R*) and liquid (*E*) *Ruminococcus*-treated groups, there was a marked increase of thymic elements. In the HS-treated group, the thymus compartment showed severe necrosis with mononuclear inflammatory cell infiltration and loss of the normal architecture of the thymic compartments. In the heat stress group treated with powder and liquid *Ruminococcus* (HS+R and HS+E), the thymic compartments showed a marked decrease of medullary depletion with a marked increase of thymocytes (Figure 5).

### Expression of Some Heat Stress-Associated Genes Within the Bursa of Fabricius

**Figure 6:**
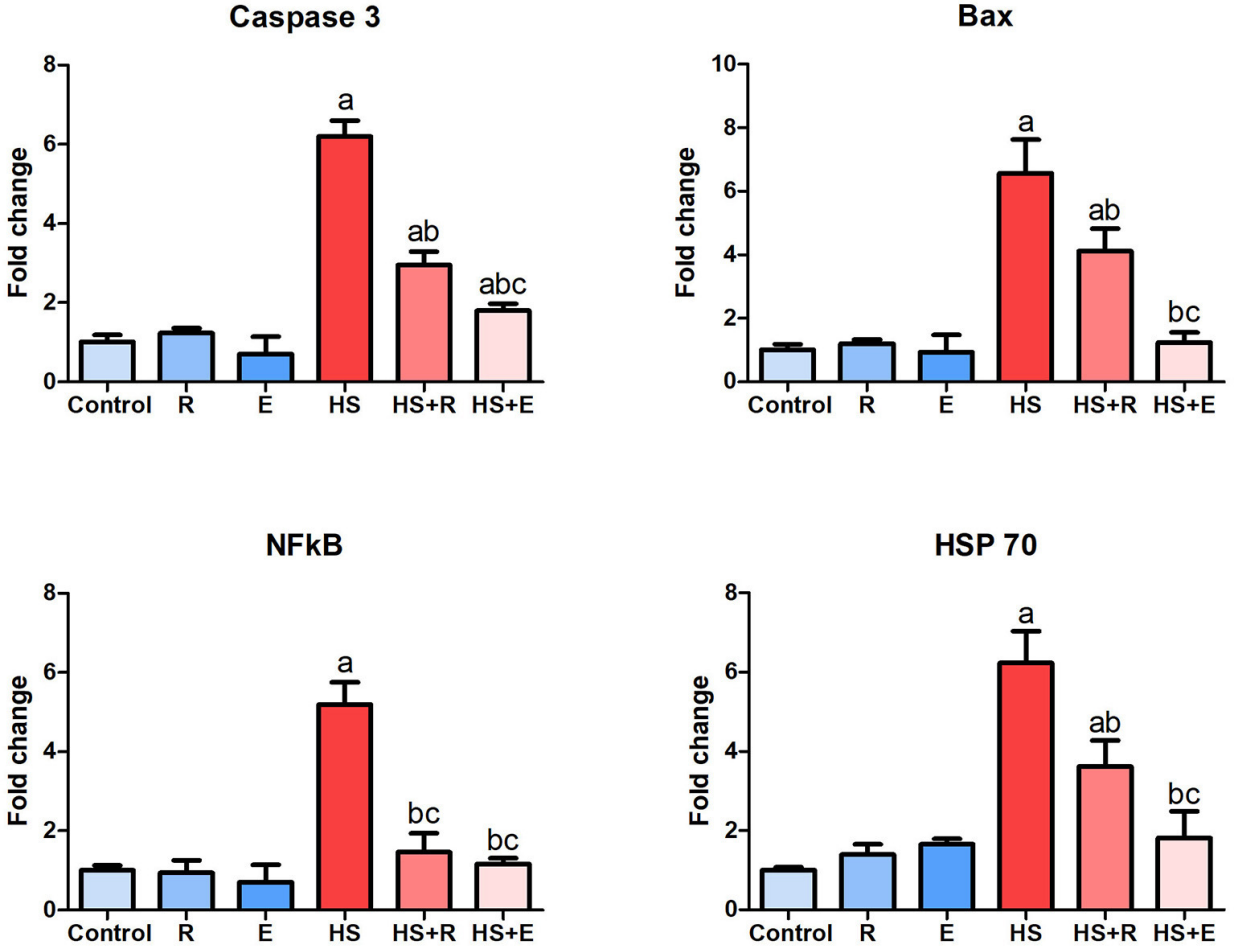
Expression of caspase 8, Bax, NF-κβ1, and HSP 70 genes within the bursa of Fabricius. Data are expressed as mean ± SD, and superscript letters a, b, and c indicate significance in comparison with the control group, HS group, and HS+R group, respectively.

The response of some genes related to heat stress, including proapoptotic genes such as caspase8 and bax, inflammatory genes such as NF-κβ1, and heat shock protein such as HSP 70, is illustrated in Figure 6. During the comfortable environment, the expression of the different genes was similar to that of the normal control group. The expression of both proapoptotic genes was markedly increased in the HS group in comparison with the control group (*P* > 0.005). A marked decrease of these genes with supplementation of *Ruminococcus* spp. as powder or liquid was relevant with the control group (*P* > 0.005). A significant decrease of both caspase 8 and bax gene expression was noticed in supplementation with the liquid form than the powder form of *Ruminococcus* (*P* >0 0.05). The bursal tissues of HS animals revealed a marked elevation of NF-κβ1 than that of the normal group (*P* > 0.005), which decreased to the normal limits of the control groups. In the same way, HSP 70 mRNA expression was markedly increased in HS than control birds and significantly decreased in heat-stressed birds supplemented with *Ruminococcus* sp. than HS birds (*P* > 0.05).

## Discussion

The results of the clinical signs in the HS group are compatible with the results of Li et al. (33) who reported that heat stress significantly affects the daily behavior of broilers, including feeding, drinking, lying, standing, and walking. Moreover, Rodrigues et al. (34) recorded a high percentage of fecal moisture as a result of heat stress. Moreover, the results of the growth were significantly affected by heat stress. These results may be attributed to heat exposure where young chicks are characterized with high metabolic rate, fast growth rate, and poor ability to adapt to environmental changes. Moreover, the chicks do not have sweat glands in their skin, so they are highly sensitive and vulnerable to heat stress (33). These results are constant with the results of Awad et al. (35) who reported that heat exposure significantly decreases feed intake and weight gain and increases FCR. Heat stress is one of the key environmental factors that affect the growth of broilers (36). On the other side, Marchini et al. (37) reported that short cycle heat does not impair the performance of the broiler. These findings were supported by the results of the gene expression within the bursa of Fabricius where immune suppression is another disadvantage of temperature modulation (38), with the results of oxidative stress that emerges from heat stress. However, the treatments significantly improved the growth parameters. These results may be attributed to the use of enzymes and/or probiotics especially (E group). These may be a result of the presence of some enzymes that digest undigested material, such as cellulase enzymes that digest cellulose. Moreover, probiotics affect the physiology and intestinal morphology of the chicks, which is indicated by the results of serum biochemistry, liver tissue antioxidant, and histopathology. Enzymatic supplementation in broilers alleviates the compromised growth and damage of the intestine induced by heat stress (39). Hosseini and Afshar (40) said that enzymatic supplementation ameliorates the resistance to heat stress in birds under high ambient temperature. Moreover, the supplementation of probiotics in the present investigation significantly improved the growth in the heat-exposed groups. These results are compatible with the results of Al-Fataftah and Abdelqader (41) and Jahromi et al. (42). On the other side, Li et al. (43) reported that probiotic supplementation could improve the morphology of the intestine and barrier function and alleviate inflammatory response but has no effect on growth under heat exposure. Moreover, Sandikci et al. (44) and Sohail et al. (45, 46) reported that probiotics have no effect on the growth of the broilers stressed with heat.

On the other side, the results of the high temperature on some carcass traits of broilers are consistent with those of Yousaf et al. (47) who reported that heat stress has deleterious effects on the carcass traits of broiler Ross-308 chicken. Moreover, Ohtsu et al. (48) recorded the negative impact of thermal temperature on immune system organs such as the spleen, which atrophied in broilers by thermal temperature. On the other side, the result of this investigation is opposite to that of Hosseini-Vashan et al. (49) who said that thermal temperature did not change the lymphoid organs' relative weight. Moreover, Rosa et al. (50) recorded that heat stress increases carcass yield and decreases heart, gizzard, and liver yield. However, these results were improved by digestive enzymes and/or *Ruminococcus sp*. supplementation. Dietary supplementation of probiotics with or without heat stress enhanced the production parameters and traits of the carcass and improved the clinical blood parameters. Moreover, Abramowicz et al. (51, 52) reported that probiotics enriched with choline improve the growth performance, immune status, histological parameters, and intestinal microbiota of broiler chickens. Thus, probiotics can be used to counteract the adverse effects of heat stress (53). de Souza et al. (54) reported that probiotics do not influence the characteristics of the carcass traits. This may be attributed to the difference in the probiotic used. Hosseini and Afshar (55) recorded the positive effect of the digestive enzymes on the production parameters, which consequently affect the carcass traits in broilers with high ambient temperature.

Regarding the results of the hematological parameters of broilers exposed to thermal stress, there was a significant decrease in red blood cell count, hemoglobin percent, and hematocrit percent. These may be due to decreased feed intake, which consequently affects the hemato-biochemical parameters of the bird through its effects on organs. Moreover, the decreased number of RBCs resulted in a decrease in hematocrit (HCT) percent. These results are in harmony with the results of Dinu et al. (56), Chaturvedani et al. (57), and Mushawwir et al. (58) who reported a significant decrease in hemoglobin and hematocrit in the broilers exposed to thermal stress. However, the supplementation of digestive enzymes and/or *Ruminococcus* sp. improved the red blood cell count, hemoglobin percent, and HCT. These results were attributed to the healthy effect of both digestive enzymes and probiotics on broilers, which appeared in the results of liver antioxidant and emerged by histopathological findings. Probiotic supplementation with heat stress conditions significantly improved the hematological measurements in broiler chicks (59). However, Sugiharto et al. (60) reported that probiotics have no effect on the chicks' hematology in normal environmental conditions. On the other side, the results of the leukogram were negatively affected under thermal stress. These results are in agreement with those of Chaturvedani et al. (57) who reported that the heat stress in broilers significantly increased heterophils, H/L ratios, and basophils. Meanwhile, the total leukocytic count and monocytes are significantly decreased, and this decrease was inconsistent with the present results. Moreover, thermal stress increases leukocytic count, heterophils, and the ratio between heterophils and lymphocytes in the brown birds (61). These adverse effects were improved by treatments, especially with enzymes and probiotic supplementation. The probiotic dietary inclusion decreases heterophils and the ratio between heterophils and lymphocyte and increases the lymphocytes in the laying hen (62).

In the heat stress group, the activities of the serum enzymes (AST, ALT, and ALP) were significantly increased. These results may be attributed to the inflammatory process that resulted from heat exposure and are indicated by histopathology. The above-mentioned result is consistent with those of Bueno et al. (63) and Huang et al. (64). Moreover, the increased environmental temperature in the present experiment adversely affects liver tissue antioxidant enzyme activities with the increment of the MDA concentration. These results are in agreement with those of Hosseini-Vashan et al. (49) who reported that thermal stress impairs the production parameters, increases the activities of AST, ALT, and ALP and the MDA concentration, and decreases antioxidant enzymes in broilers. The increased activities of ALT, AST, and ALP indicate liver injury (65), which was supported by histopathological findings. The probiotic dietary inclusion decreased the serum activities of ALT, AST, and ALP in the laying hen (62). Other findings reported that probiotics dietary supplementation without heat stress does not affect the serum activities of AST and ALT (66). These results have been confirmed by the results of MDA and antioxidant enzymes.

The results of the liver, bursa, and spleen weights, growth performance, tissue antioxidant, and pathology are consistent with the results of the phagocytic assay. The result of the phagocytic assay is compatible with those of Laganá et al. (67), Quinteiro-Filho et al. (68), and Dalólio et al. (69) who reported that high temperature promotes a decrease in phagocytic activity. Moreover, Attia et al. (70) recorded the adverse effect of heat stress on phagocytic assay in broilers. These adverse effects were mitigated by using the supplements, and it has been confirmed by Attia et al. (70) and Alkhalf et al. (71) who indicated the valuable effects of probiotics on broilers' immune system with and without heat stress, respectively. Kidd (72) demonstrated that enzymes enhance feed digestion and nutrient absorption that, in turn, can affect body immunity.

On the other hand, the results of the histopathology confirm and support previous results. In the heat-stressed birds, the result of the liver pathology is in agreement with that of Aengwanich and Simaraks (73), wherein liver cells exhibited fatty degeneration with dilation of all broilers' sinusoids. Moreover, in some parts of the liver, especially in the central rhythmic region, necrosis was observed with heterophils and lymphocytes. Anju Rajan et al. (74) observed that intra-follicular and intra-epithelial cysts were present in the bursa of the heat-stressed broilers. Moreover, Hirakawa et al. (75) reported that heat stress seriously impaired the morphology of thyme and bursal follicles, which was confirmed by the result of the phagocytic assay. On the other hand, the supplementation of probiotics alone or with enzymes improved the pathology of the internal organs. These results were confirmed by the result of Shah et al. (76) wherein probiotic supplementation improves the immune organs' histomorphology. These improvements in the pathology of the internal organs may be attributed to the antioxidant effect of probiotics.

On a molecular basis, it was noticed that heat stress increased the apoptotic biomarkers including caspase 8 and bax genes. A marked elevation of inflammatory genes was also demonstrated, while the HSP genes' level of expression was variable according to the species and the duration of heat exposure. Our previous studies on hepatic and splenic tissues revealed a marked elevation of the different biomarkers such as heat shock proteins, inflammatory, and apoptotic genes (26, 27, 32–77).

Herein we selected the bursal tissues to explore the noticeable early signs of bursal involution, which indicated the presence of intra-follicular mucosal cysts and interstitial fibrosis. The early bursal atrophy was correlated well with the marked upregulation of apoptotic genes, including bax and caspase 8. On the other hand, HPPs are considered one of the cellular homeostasis mechanisms against heat stress. Experimental studies reported the upregulation of HSPs in chronic heat conditioning in a time-dependent manner.

As molecular chaperones, heat shock proteins play a crucial role by helping to correctly pliage and avoid the aggregation of emerging and stress-accumulated misplant proteins that have a defensive feature, which allows the cells to survive under conditions otherwise considered lethal (78). The result of gene expression in the bursal tissue of heat-stressed broiler is in agreement with the result of Giffard et al. (79) where the expression of both proapoptotic genes was markedly increased in the HS group. Moreover, Akbarian et al. (80) reported that the activity and the regulation of NF-κβ1 are affected by thermal temperature.

## Conclusion

The present experiment demonstrated that the administration of a probiotic with or without enzymes throughout the rearing period of the broiler chicks with or without heat stress had the most beneficial effect on growth performance, enzymatic activity of serum liver, liver tissue antioxidants and peroxide, and phagocytic assay. Moreover, the supplements improved the pathological lesions in the internal organs that resulted from heat stress.

## Data Availability Statement

The datasets used and/or analyzed during the current study are available from corresponding author on reasonable request.

## Ethics Statement

The animal study was reviewed and approved by Research Ethical Committee of the Faculty of Veterinary Medicine, Kafrelsheikh University, Egypt (Approval No. KFS-2019-12).

## Author Contributions

All authors designed and conducted the experiment, wrote the manuscript, read, and approved the manuscript.

## Conflict of Interest

The authors declare that the research was conducted in the absence of any commercial or financial relationships that could be construed as a potential conflict of interest.

## Correction note

A correction has been made to this article. Details can be found at: 10.3389/fvets.2026.1838754.
