# Correction: Impact of dietary or drinking water *Ruminococcus* sp. supplementation and/or heat stress on growth, histopathology, and bursal gene expression of broilers

**DOI:** 10.3389/fvets.2026.1838754

**Published:** 2026-05-07

**Authors:** Adel Hassan Saad, Mohamed S. Ahmed, Mohamed Aboubakr, Hanan A. Ghoneim, Mohamed M. Abdel-Daim, Ghadeer M. Albadrani, Nagah Arafat, Sabreen Ezzat Fadl, Walied Abdo

**Affiliations:** 1 Nutrition and Clinical Nutrition Department, Faculty of Veterinary Medicine, Matrouh University, Mersa Matruh, Egypt; 2 Pathology Department, Faculty of Veterinary Medicine, Kafrelsheikh University, Kafr El-Shaikh, Egypt; 3 Pharmacology Department, Faculty of Veterinary Medicine, Benha University, Banha, Egypt; 4 Department of Physiology, Faculty of Veterinary Medicine, Damanhour University, Damanhour, Egypt; 5 Pharmacology Department, Faculty of Veterinary Medicine, Suez Canal University, Ismailia, Egypt; 6 Department of Biology, College of Science, Princess Nourah bint Abdulrahman University, Riyadh, Saudi Arabia; 7 Department of Poultry Diseases, Faculty of Veterinary Medicine, Mansoura University, Mansoura, Egypt; 8 Biochemistry Department, Faculty of Veterinary Medicine, Matrouh University, Mersa Matruh, Egypt

**Keywords:** heat stress, *Ruminococcus*, enzyme, biochemistry, phagocytic assay, pathology, bursal gene

There was a mistake in Figure 5 as published. Figure 5E was accidentally replaced by another image that belongs to a different group of the same experiment. The corrected version of figure number 5 and its caption appear below.

**Figure 1:**
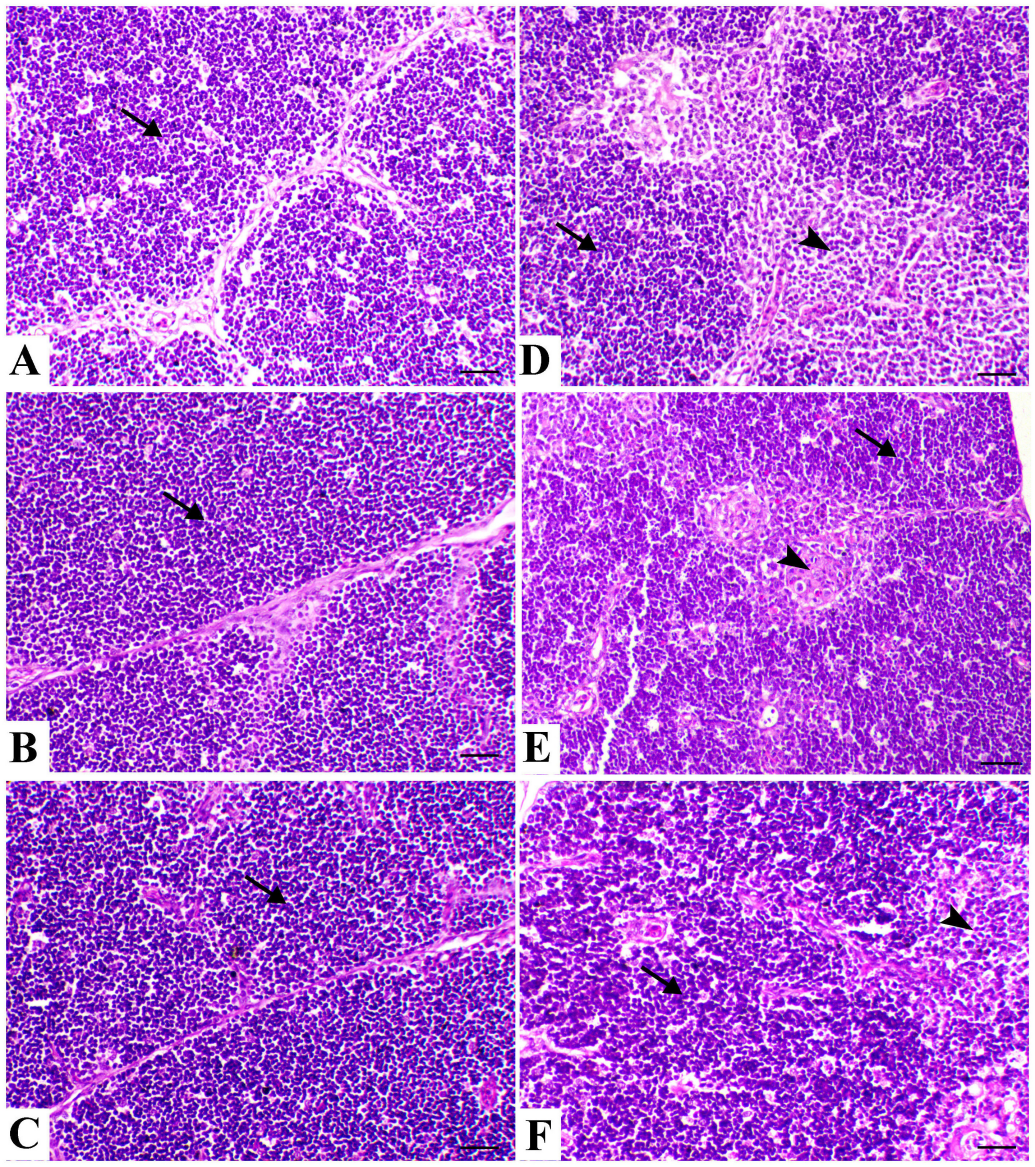
Thymus sections of the different treated groups. **(A)** Thymus of the control group showing normal thymic compartments (the arrow indicates normal thymocytes). Sham groups **(B)** R and **(C)** E revealing normal thymic compartments. **(D)** Spleen of the heat stress (HS) group showing marked thymic depletion (arrow). **(E)** Spleen of HS+R showing medullary lymphoid depletion (arrowhead). **(F)** Spleen of HS+E showing a marked restoration of lymphoid content within the follicle. H&E, ×200, bar = 50 μm.

The original version of this article has been updated.

